# Combined Effect of Biological and Organic Fertilizers on Agrobiochemical Traits of Corn (*Zea mays* L.) under Wastewater Irrigation

**DOI:** 10.3390/plants13101331

**Published:** 2024-05-12

**Authors:** Hossein Shirzad, Sina Siavash Moghaddam, Amir Rahimi, Salar Rezapour, Jianbo Xiao, Jelena Popović-Djordjević

**Affiliations:** 1 Department of Plant Production and Genetics, Faculty of Agriculture, Urmia University, Urmia 5756151818, Iran; shirzadhossein93@gmail.com (H.S.); emir10357@gmail.com (A.R.); 2 Department of Soil Science, Faculty of Agriculture, Urmia University, P.O. Box 165, Urmia 57134, Iran; s_rezapour2000@yahoo.com; 3 Department of Analytical Chemistry and Food Science, Faculty of Food Science and Technology, University of Vigo, 36310 Vigo, Spain; jianboxiao@uvigo.es; 4 Faculty of Agriculture, University of Belgrade, Nemanjina 6, 11080 Belgrade, Serbia

**Keywords:** corn, antioxidants, cadmium, biochar, glomalin, organic and biofertilizers, wastewater

## Abstract

Corn (*Zea mays* L.) is an important annual grain that is cultivated as a food staple around the world. The current study examined the effect of wastewater and a combination of biological and organic fertilizers on the morphological and phytochemical traits of corn, using a factorial experiment based on a randomized complete block design with three replications. The first factor was biological and organic fertilizers at seven levels, including the control (no fertilization), bacterial biological fertilizers (NPK) along with iron and zinc Barvar biofertilizers, fungal biofertilizers made from Mycorrhiza and *Trichoderma*, biochar, a combination of bacterial and fungal biofertilizers, and a combination of bacterial and fungal biofertilizers with biochar. The second factor was irrigation at two levels (conventional irrigation and irrigation with wastewater). The traits studied included the morphological yield, phenols, flavonoids, polyphenols, glomalin, cadmium content in plant parts, and translocation factor (TF). The results disclosed that the best treatment in regard to the morphological traits was related to conventional water + biochar + mycorrhiza + *Trichoderma* + NPK. The highest phenol and flavonoid content were observed when biochar + mycorrhiza + *Trichoderma* + NPK treatments were used in both water treatments. Also, the wastewater + biochar + mycorrhiza + *Trichoderma* + NPK treatment demonstrated the highest total glomalin and phenylalanine ammonia-lyase (PAL) activity. The obtained results demonstrate that combined biological and organic fertilizer use on corn plants can effectively alleviate the deleterious effects of cadmium present in wastewater.

## 1. Introduction

The preservation of both the quantity and quality of product from the agricultural industry is crucial for the sustenance of nations and the success of economic exports. As such, agriculture has undergone various scientific innovations to improve its efficiency [1]. According to the Food and Agriculture Organization, the world population will be 9 billion by 2050. So, crop yields must be increased to meet the food requirement. Soil is an important aspect of food production, which helps to sustain human life. However, it has been degraded by intensive farming practices, such as the use of pesticides and chemical fertilizers on a global scale, and it has lost its fertility due to the degradation of biodiversity, the trapping of water, and the disruption of biogeochemical cycles in recent decades. Soil health and plant productivity are severely affected by various interactions between plants, soil, and microorganisms [2]. Among the available crops, corn (*Zea mays* L.) is one of the most diverse crops, with high genetic potential and extensive adaptation to different agroclimatic conditions [3]. It is the source of numerous food and industrial products [4,5,6]. Corn is known as the “queen” of cereals around the world, as it has been cultivated in over 170 countries, with a total cereal production rate of 1219.76 million tons [7]. 

Water demand is continuously growing in arid and semi-arid countries. Thus, water with higher quality is reserved for domestic uses and that with lower quality is recommended for irrigation [8]. The utilization of domestic wastewater for irrigation purposes serves as a safe and effective method of wastewater disposal, ultimately aiding in the reduction of pollution in ecosystems that would otherwise be impacted by direct wastewater discharge into surface or underground water sources [9,10]. Wastewater may contain agriculturally beneficial nutrients. However, its continuous application may increase potentially toxic elements in plants and soils [11]. Wastewater may also contain industrial waste, potentially toxic elements, contaminated soil, and food waste [12]. The investigation of crop resistance to pollution by potentially toxic elements and the locus of their accumulation in plant tissues is necessary for understanding how dangerous they are to human health [13,14]. The exposure of plants to Cd causes the formation of reactive oxygen species (ROS), which induces oxidative stress and the formation of several types of cell damage, such as the increased peroxidation of lipids, the accumulation of H_2_O_2_, changes in the expression of several genes, and the modulation of the antioxidant system [15]. 

Researchers have recently become interested in the use of soil amendments, for e.g., biochar, to immobilize metals that are present in soil solutions and reduce their uptake by plants [16,17]. Hydrothermal carbonization is a suitable strategy for the carbonization of wet materials. The advantages of using this method include the mild temperature, reduced production of toxic gases, no need to dry the wet biomass, and the use of water as a solvent and catalyst. The hydrochar obtained using this method is a solid product with a brown–black color and it is rich in carbon [18,19]. Microwave hydrothermal carbonization is a route to convert seaweed into value-added products, such as hydrochar [20]. Algal biomass is converted into hydrochar by the hydrothermal method, which depends on factors such as the reaction temperature, time, biomass particle size, and the amount of catalyst and biomass needed for the maximum hydrochar yield [20,21,22]. Researchers have used *Laminaria digitata* alga to produce hydrochar. The hydrochar produced from this alga had a higher calorific value comparable to coal [20]. *Trichoderma* is a genus of filamentous fungi, extensively used in agriculture as a growth-promoting factor [23]. Soil pollution affects the concentration of micronutrients, such as zinc, iron, copper, and manganese, which are necessary for plant growth [24]. *Trichoderma* species are resistant to potentially toxic metals, including cadmium, copper, mercury, zinc, and lead [25,26]. These fungi genus species can produce plant hormones and increase plant growth, for this reason, they are known as biofertilizer fungi. The *Trichoderma* species, isolated from the olive rhizosphere soil in northern Algeria, have shown biomolecule production of plant growth-stimulating molecules, such as phytohormones, hydrolytic enzymes, siderophores, ammonia, and phosphatases. The native strain T11 (*Trichoderma harzianum*) was used as a biofertilizer due to its ability to produce a high amount of indole-3-acetic acid (IAA) and its promising plant growth-promoting properties [27]. The effective application of soil microorganisms (plant growth-promoting rhizobacteria: PGPR) can help increase plant growth in agriculture. Different genera of *Pseudomonas* and *Azotobacter* are PGPRs that enhance plant growth in both normal and stressful conditions [28]. Recently, it has been reported that marine macroalgae are being used as plant biostimulants. Species of these macroalgae have shown the ability to produce gibberellic acid (*Hypnea corona* Huisman et Petrocelli (*Rhodophyta*)), indoleacetic acid, abscisic acid, kinetin, and indole butyric acid (*Spyridia filamentosa* (Wulfen) Harvey (*Rhodophyta*)). These macroalgae are abundant in food-rich coastal environments and act as a nuisance to human activities and aquaculture. Therefore, this unwanted biomass is a specific product that could be useful from the point of view of sustainable environmental development and sustainable agriculture [29]. 

Phenolic and flavonoid compounds are bioactive substances commonly present in various plant species. Studies have demonstrated that these compounds possess notable antioxidant properties [30]. Several antioxidant mechanisms have been reported in these compounds, such as ROS scavenging, metal chelation, increased endogenous antioxidant enzyme activity, and suppression of ROS-synthesizing prooxidant enzymes [31,32]. Glomalin is an *N*-linked glycoprotein that is considered a product of the gene AMF (arbuscular mycorrhizal fungi) and is defined as a protein that is secreted by AMF hyphae and spores [33,34].

Phenylalanine ammonia-lyase (PAL) is an enzyme that is considered to be the interface between initial and secondary metabolism. It is also one of the main lines of cell adaptation to various biotic and abiotic stresses involving potentially toxic elements [35]. It has also been revealed that PAL is a critical compound in induced systemic resistance (ISR) and salicylic acid (SA) synthesis and an essential signal in ISR [36]. It plays a fundamental role in the synthesis of several defense-related secondary compounds, such as phenols and lignin [37,38]. The translocation of metals from the soil to plant tissues is assessed by an index called the translocation factor (TF), which is defined as the ratio of the concentration of a certain metal in plant tissue to its concentration in the soil [39].

Potentially toxic elements naturally occur in the earth [40], but industrial and agricultural activities pertaining to chemical fertilizers, wastewater, and so on, may increase their accumulation in the soil and exert long-term effects on ecosystems [41,42,43]. Furthermore, these metals may impact human health because people may consume crops produced in regions polluted with potentially toxic elements [44,45,46].

Given the current climatic conditions in the world and water scarcity, it seems unavoidable that unconventional water resources will need to be consumed. The present work aims to raise farmers’ awareness of the possible benefits and dangers of using wastewater, and how to reduce the negative effects of potentially toxic metals contained in these water sources by using organic fertilizers and biofertilizers in corn fields.

## 2. Results

### 2.1. Cob Length

**Figure 1:**
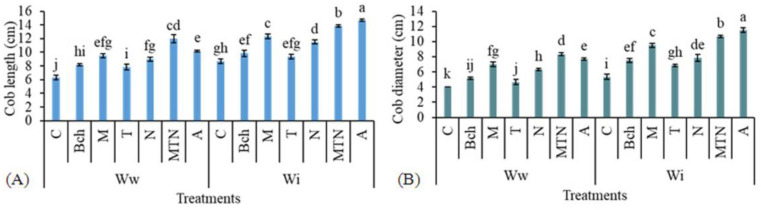
A comparison of the means for cob length (cm) (**A**) and cob diameter (cm) (**B**) in plants treated with Ww (wastewater), Wi (conventional water), and fertilizers, which include C (control), Bch (biochar), M (mycorrhiza), T (*Trichoderma)*, N (NPK), MTN (mycorrhiza + *Trichoderma* + NPK), A (biochar + mycorrhiza + *Trichoderma* + NPK). Various letters above the bars indicate notable differences at a significance level of 5% according to Duncan’s test.

The longest cobs were observed in the plants treated with conventional water + biochar + mycorrhiza + *Trichoderma* + NPK (WiA) and the shortest were related to those treated with pure wastewater (Wwc). There was a significant difference between the treatment with conventional water + biochar + mycorrhiza + *Trichoderma* + NPK (WwMTN) and the treatment with wastewater + biochar + mycorrhiza + *Trichoderma* + NPK (WwA). The cob length was the highest in the plants treated with WwMTN, but it did not significantly differ from the treatments with wastewater + mycorrhiza (WwM) and wastewater + NPK (WwN) (Figure 1A).

### 2.2. Cob Diameter

The plants treated with conventional water + biochar + mycorrhiza + *Trichoderma* + NPK (WiA) exhibited the largest cob diameter. The lowest diameter was found using pure wastewater (Wwc). There was a significant difference between the treatments with conventional water + biochar + mycorrhiza + *Trichoderma* + NPK (WiA) and wastewater + biochar + mycorrhiza + *Trichoderma* + NPK (WwA). The plants treated with wastewater + mycorrhiza + *Trichoderma* + NPK (WwMTN) had the largest cob diameter, significantly different from all the wastewater treatments (Figure 1B).

### 2.3. Number of Grains per Row

**Figure 2:**
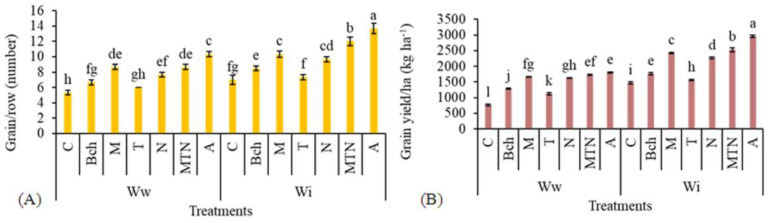
A comparison of means for the number of grains per row (**A**) and grain yield (kg/ha) (**B**) in plants treated with Ww (wastewater), Wi (conventional water), and fertilizers, which include C (control), Bch (biochar), M (mycorrhiza), T (*Trichoderma)*, N (NPK), MTN (mycorrhiza + *Trichoderma* + NPK), A (biochar + mycorrhiza + *Trichoderma* + NPK). Various letters above the bars indicate notable differences at a significance level of 5% according to Duncan’s test.

The plants treated with conventional water + biochar + mycorrhiza + *Trichoderma* + NPK (WiA) had the highest number of grains per row and those treated with pure wastewater (Wwc) had the least. A significant difference was detected between the treatments with conventional water + biochar + mycorrhiza + *Trichoderma* + NPK (WiA) and wastewater + biochar + mycorrhiza + *Trichoderma* + NPK (WwA). The plants treated with wastewater + biochar + mycorrhiza + *Trichoderma* + NPK (WwA) produced the highest number of grains per row. This treatment significantly differed from the other treatments, but no significant difference was observed between the treatments with wastewater + mycorrhiza (WwM) and wastewater + mycorrhiza + *Trichoderma* + NPK (WwMTN) (Figure 2A).

### 2.4. Grain Yield per ha

The highest grain yield per hectare was observed in the treatment with conventional water + biochar + mycorrhiza + *Trichoderma* + NPK (WiA) and the lowest was observed in the treatment with wastewater (Wwc). The treatment with conventional water + biochar + mycorrhiza + *Trichoderma* + NPK (WiA) showed a significant difference in regard to this trait from the treatment with wastewater + biochar + mycorrhiza + *Trichoderma* + NPK (WwA). In the plants treated with wastewater and biochar + mycorrhiza + *Trichoderma* + NPK (WwA), the grain yield per ha was the highest, but did not differ significantly from the treatment with wastewater and mycorrhiza + *Trichoderma* + NPK (WwMTN) (Figure 2B).

### 2.5. Antioxidant Indices

#### 2.5.1. Leaf Phenols

**Figure 3:**
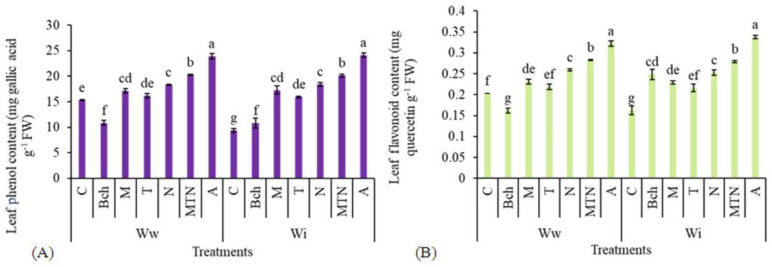
A comparison of means for leaf phenol content (mg gallic acid g^−1^ FW) (**A**) and leaf flavonoid content (mg quercetin g^−1^ FW) (**B**) in plants treated with Ww (wastewater), Wi (conventional water), and fertilizers, which include C (control), Bch (biochar), M (mycorrhiza), T (*Trichoderma)*, N (NPK), MTN (mycorrhiza + *Trichoderma* + NPK), A (biochar + mycorrhiza + *Trichoderma* + NPK). Various letters above the bars indicate notable differences at a significance level of 5% according to Duncan’s test.

The leaf phenols exhibited the highest values when conventional water + biochar + mycorrhiza + *Trichoderma* + NPK (WiA) was used as the treatment, whereas conventional water (Wic) was associated with the lowest values of leaf phenols. There was not a significant difference between the treatment with conventional water + biochar + mycorrhiza + *Trichoderma* + NPK (WiA) and the treatment with wastewater + biochar + mycorrhiza + *Trichoderma* + NPK (WwA). The plants treated with wastewater + mycorrhiza (WwM) did not differ significantly from those treated with wastewater + NPK (WwN) (Figure 3A).

#### 2.5.2. Leaf Flavonoids

The highest leaf flavonoid was related to the treatment with conventional water + biochar + mycorrhiza + *Trichoderma* + NPK (WiA) and the lowest related to the treatment with conventional water (Wic). However, the latter treatment did not differ significantly from the wastewater + biochar (WwBch) treatment. The treatment with wastewater + biochar + mycorrhiza + *Trichoderma* + NPK (WwA) significantly increased this trait versus the irrigation with wastewater. No significant difference was observed between the treatment with conventional water + biochar + mycorrhiza + *Trichoderma* + NPK (WiA) and the treatment with wastewater + biochar + mycorrhiza + *Trichoderma* + NPK (WwA). The plants treated with mycorrhiza + *Trichoderma* + NPK (WwMTN and WiMTN) were not significantly influenced by the irrigation treatments (Figure 3B).

#### 2.5.3. Polyphenolic Profile

The assay of polyphenols using gallic acid showed that the treatment with wastewater + NPK (WwN) had the highest value of polyphenols and the treatment with wastewater + *Trichoderma* (Wwt) had the lowest value (Figure S1a). The caffeic acid content varied among the treatments, with the highest levels observed in the conventional water + biochar treatment (WiBch), and the lowest levels observed in the conventional water + NPK treatment (WiN) (Figure S1b). Chlorogenic acid and quercetin were the highest in the treatments with wastewater + mycorrhiza (WwM) and wastewater + biochar + mycorrhiza + *Trichoderma* + NPK (WwA) and the lowest in the treatment with wastewater + NPK (WwN) (Figure S2a,b). The treatments with wastewater + biochar + mycorrhiza + *Trichoderma* + NPK (WwA) and conventional water + biochar (WiBch) exhibited the highest and lowest rutin content, respectively (Figure S3a). Coumaric acid was the highest in the treatment with wastewater + mycorrhiza + *Trichoderma* + NPK (WwMTN) and the lowest in the treatment with conventional water + mycorrhiza (WiM) (Figure S3b). Rosmarinic acid was the highest in the treatment with conventional water + *Trichoderma* (WiT) and the lowest in the treatment with wastewater + NPK (WwBch) (Figure S4a). The treatments with wastewater (Wwc) and conventional water (Wic) yielded the highest levels of cinnamic acid and apigenin content, while the treatment involving wastewater + biochar (WwBch) resulted in the lowest content of these compounds (Figure S4b,c).

### 2.6. Simple Glomalin

**Figure 4:**
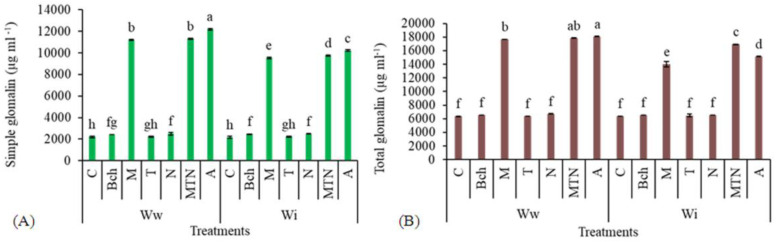
A comparison of means for simple glomalin (µg ml^−1^) (**A**) and total glomalin (µg ml^−1^) (**B**) in plants treated with Ww (wastewater), Wi (conventional water), and fertilizers, which include C (control), Bch (biochar), M (mycorrhiza), T (*Trichoderma)*, N (NPK), MTN (mycorrhiza + *Trichoderma* + NPK), A (biochar + mycorrhiza + *Trichoderma* + NPK). Various letters above the bars indicate notable differences at a significance level of 5% according to Duncan’s test.

The highest simple glomalin was observed in the treatment with wastewater + biochar + mycorrhiza + *Trichoderma* + NPK (WwA) and the lowest in the treatment with conventional water (Wic), which did not differ significantly from the treatments with wastewater (Wwc), wastewater + *Trichoderma* (Wwt), and conventional water + *Trichoderma* (WiT). The treatment with wastewater + biochar + mycorrhiza + *Trichoderma* + NPK (WwA) had significantly increased simple glomalin versus its lowest value in the irrigation with wastewater. No significant difference was observed between the treatment with conventional water + mycorrhiza + *Trichoderma* + NPK (WwMTN) and the treatment with wastewater + mycorrhiza (WwM) (Figure 4A).

### 2.7. Total Glomalin

The treatment with wastewater + biochar + mycorrhiza + *Trichoderma* + NPK (WwA) exhibited the highest total glomalin, but it did not differ significantly from the treatment with conventional water + mycorrhiza + *Trichoderma* + NPK (WwMTN). The lowest total glomalin was associated with the treatment with wastewater (Wwc), which did not differ significantly from the treatments with wastewater + biochar (WwBch), wastewater + *Trichoderma* (Wwt), wastewater + NPK (WwN), conventional water (Wic), conventional water + biochar (WiBch), conventional water + mycorrhiza (WiM), conventional water + *Trichoderma* (WiT), and conventional water + NPK (WiN) (Figure 4B).

### 2.8. Phenylalanine Ammonia-Lyase (PAL)

**Figure 5:**
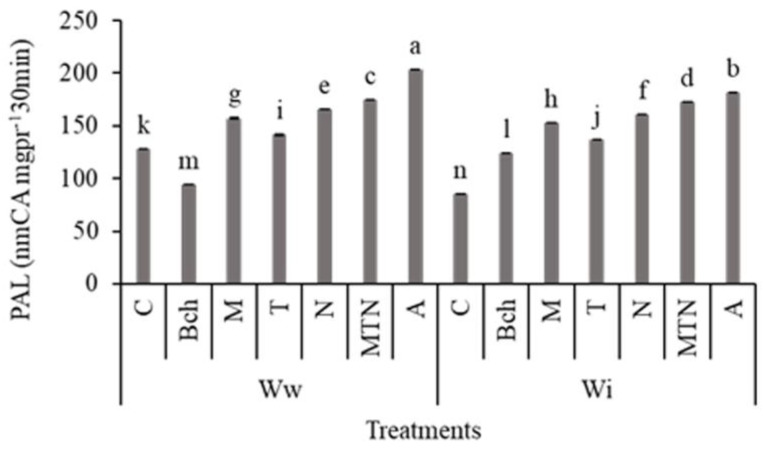
A comparison of means for phenylalanine ammonia-lyase (PAL) (nmCA mg pr^−1^ 30 min) activity in plants treated with Ww (wastewater), Wi (conventional water), and fertilizers, which include C (control), Bch (biochar), M (mycorrhiza), T (*Trichoderma)*, N (NPK), MTN (mycorrhiza + *Trichoderma* + NPK), A (biochar + mycorrhiza + *Trichoderma* + NPK). Various letters above the bars indicate notable differences at a significance level of 5% according to Duncan’s test.

The results showed that the highest PAL activity was related to the wastewater + mycorrhiza + *Trichoderma* + NPK (WwMTN) treatment, and the wastewater + biochar + mycorrhiza + *Trichoderma* + NPK (WwA) treatment, which differed significantly from the other treatments. The lowest activity was observed in the treatment with conventional water (Wic). It also differed significantly from the other treatments (Figure 5).

### 2.9. Cadmium Content in Root and Grain

**Figure 6:**
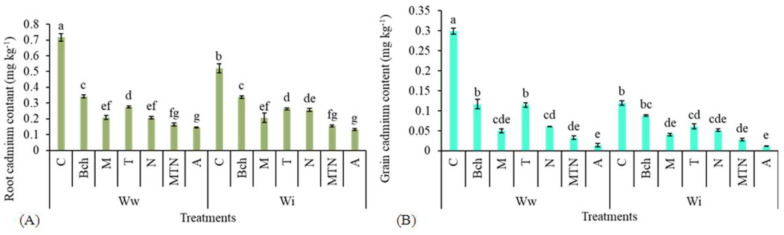
A comparison of means for root cadmium content (mg kg^−1^) (**A**) and grain cadmium content (mg kg^−1^) (**B**) in plants treated with Ww (wastewater), Wi (conventional water), and fertilizers, which include C (control), Bch (biochar), M (mycorrhiza), T (*Trichoderma)*, N (NPK), MTN (mycorrhiza + *Trichoderma* + NPK), A (biochar + mycorrhiza + *Trichoderma* + NPK). Various letters above the bars indicate notable differences at a significance level of 5% according to Duncan’s test.

The highest and lowest root cadmium content were obtained in the treatments with wastewater (Wwc) and conventional water + biochar + mycorrhiza + *Trichoderma* + NPK (WiA). There was no significant difference between the treatments with conventional water + biochar + mycorrhiza + *Trichoderma* + NPK (WiA), wastewater + biochar + mycorrhiza + *Trichoderma* + NPK (WwA), and wastewater + mycorrhiza + *Trichoderma* + NPK (WwMTN) (Figure 6A).

The treatment with wastewater (Wwc) was related to the highest grain cadmium content and the treatment with conventional water + biochar + mycorrhiza + *Trichoderma* + NPK (WiA) was related to the lowest grain cadmium content. There was no significant difference between the treatments with wastewater + biochar + mycorrhiza + *Trichoderma* + NPK (WwA), water + biochar + mycorrhiza + *Trichoderma* + NPK (WiA), wastewater + mycorrhiza + *Trichoderma* + NPK (WwMTN), and conventional water + mycorrhiza + *Trichoderma* + NPK (WiMTN) (Figure 6B).

### 2.10. Translocation Factor (TF) from the Roots to the Grains

**Figure 7:**
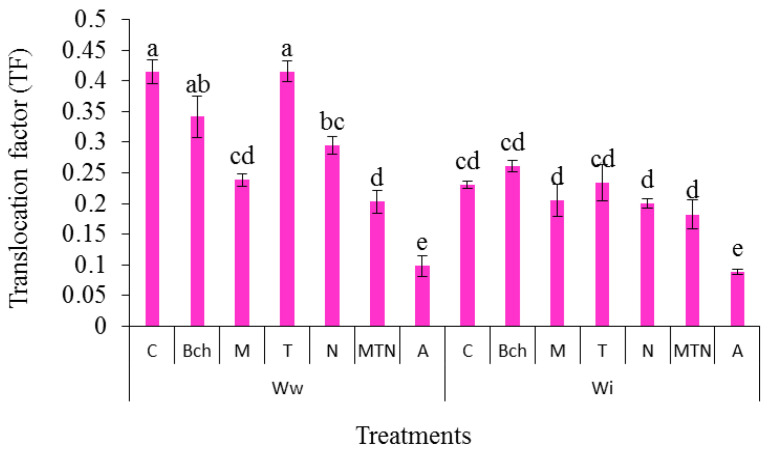
A comparison of means for translocation factor (TF) in plants treated with Ww (wastewater), Wi (conventional water), and fertilizers, which include C (control), Bch (biochar), M (mycorrhiza), T (*Trichoderma)*, N (NPK), MTN (mycorrhiza + *Trichoderma* + NPK), A (biochar + mycorrhiza + *Trichoderma* + NPK). Various letters above the bars indicate notable differences at a significance level of 5% according to Duncan’s test.

The results on the TF from the roots to the grains showed that the highest was exhibited in the samples from those given the conventional wastewater (Wwc) treatment and the lowest by the conventional water + biochar + mycorrhiza + *Trichoderma* + NPK (WiA) treatment. There was no significant difference between the treatments with wastewater (Wwc), wastewater + biochar (WwBch), and wastewater + *Trichoderma* (WwT) (Figure 7).

### 2.11. ANOVA of Morphological and Biochemical Traits

According to the analysis of variance (ANOVA), the morphological and biochemical traits of the corn were significantly influenced at the *p* < 0.01 and *p* < 0.05 levels (Table 1).

## 3. Discussion

Based on the results, the best yield was related to conventional water combined with all the fertilizers, which could be expected. The results for the plants irrigated with wastewater and treated with fertilizers (WwMTN and WwA) implies that the fertilizers used in this research can partially moderate the toxic effects caused by cadmium present in the wastewater used.

It has been reported that wastewater may contribute to plant growth owing to its high nutrient content. Nevertheless, its hazardous consequences can pose a significant threat to the entire food chain, as these harmful substances can accumulate in plants through water absorption [47]. Wastewater irrigation can be used as a source of organic matter to improve the physical and chemical characteristics of soil, as a potential source of nutrients. However, the main drawback of wastewater is the accumulation of immobile, potentially toxic elements in the soil. Metal toxicity and the risk of their accumulation in plants can be reduced by soil and a mixture of organic matter [8]. Another piece of research showed a decrease in the dry biomass of lettuce plants by applying 1 to 50 µM of Cd(NO_3_)_2_ [48]. A decrease was also observed in the fresh shoot biomass of fenugreek with the application of cadmium [49]. In another study, the irrigation of *Leucaena leucocephala* with polluted water reduced plant growth and height [50]. The results showed that wastewater significantly reduced the growth of quinoa versus fresh water, which was attributed to the potentially toxic elements in the wastewater, such as cadmium and lead [51]. This supports the findings in the present study.

Researchers have reported that the application of arbuscular mycorrhiza (AM) and other biofertilizers reduced the rate of mineral fertilizer application and significantly increased corn growth, yield, and quality [52]. Arbuscular mycorrhizal fungi symbiosis is probably the most extensive beneficial interaction between plants and microorganisms [53]. Several studies have shown that mycorrhiza plays an essential role in plant nutrition and growth and reinforces several essential processes in the ecosystem [54,55]. Based on the results, by expanding the root uptake area, AMF increases the total absorption area in inoculated plants, thereby improving the plant’s access to nutrients, especially those whose ions are lowly mobile or those that occur in the soil solution at low concentrations, as supported by Smith and Read [56]. It has been stated that inoculation with AMF positively affects plant growth traits [57]. Mycorrhiza symbiosis reportedly improves plant growth, root biomass, and shoot nutrient uptake, chlorophyll, carotene, protein and sugar contents, and antioxidant enzyme activities, when exposed to potentially toxic elements [58,59], which agrees with our findings.

The modification of potentially toxic elements by mycorrhiza fungi can happen through the “metallic bond” of hyphae, which reduces the bioavailability of such elements as copper, lead, cobalt, cadmium, and zinc, as also confirmed by Audet and Charest [60]. The alleviation of metal toxicity by mycorrhiza depends on the plant growth conditions, the potentially toxic elements, and their concentration [61]. Eissa and Abeed [62] reported that the presence of lead, copper, and cadmium at toxic concentrations in the tissues of quinoa caused the plant to spend a significant fraction of its energy on vital processes that reduce the adverse effect of metal toxicity, so the plants showed stunted growth. Potentially toxic elements, for e.g., lead, copper, and cadmium, harness some physiological processes and injure cell membranes, thereby minimizing plant growth [63]. The obtained results showed that in the presence of cadmium, the application of biofertilizers can be effective in preventing a decline in vital processes by providing nutrients.

Based on the results on the effect of *Trichoderma* and cadmium on antioxidant activity, the increased activity of antioxidants in the samples can be attributed to the fact that the maximum physiological activity and interaction between *Trichoderma* and the plant happen in the roots. It has also been stated that all anti-radical compounds, for e.g., phenols, flavonoids, and antioxidants, increased in plants treated with *Trichoderma* [64]. It was also observed that an increase in antioxidants and phenols occurred as a result of the *Trichoderma* treatments. The ROS scavenging pathways are generally plant specific and depend on the accumulated metal and stress severity. Phenol compounds, which are a part of numerous groups of secondary metabolites in plants, are examples of non-enzymatic components [65]. In these molecules, those that have a flavonoid structure are important antioxidants in plants due to their ability to induce reduction reactions, chelate metals, suppress enzymes, and absorb free radicals [66]. A study revealed that *Trichoderma* inoculation led to an increase in resistance against cadmium stress through the enhancement of low molecular weight antioxidant metabolism. This was evidenced by an elevation in phytochelatin, reduced glutathione, and reduced ascorbic acid levels, in conjunction with high cadmium concentrations, triggering the activation of secondary metabolite pathways. This activation resulted in the overproduction of phenols, flavonoids, and anthocyanin content [67]. The interaction of plants with environmental stress factors, including metal ions, activates a multifaceted defensive system that causes quantitative and qualitative changes in metabolite production, including polyphenols [68,69]. Flavonoid content can be increased by biological fertilizers or a combination of them [70]. Fertilization sources are an important factor in improving stress resistance, especially against abiotic stresses [71]. It was reported that the application of biofertilizers and *Trichoderma* increased polyphenols in stressful conditions [72]. Hydroxycinnamic acids (including *p*-coumaric, sinapic, caffeic, and chlorogenic acids) are the precursors for lignin synthesis [73]. The increase in phenolic compounds in mycorrhizal plants may be due to the increased absorption of nutrients and the beneficial effects on plant growth and photosynthesis. Phenolic compounds are produced in plants through malonic acid and shikimic acid pathways, which use precursors of glycolysis and the pentose phosphate pathway [74]. Arbuscular mycorrhizal fungi lead to increased activity of phenylalanine ammonia-lyase (PAL), glucose-6-phosphate dehydrogenase, and shikimate dehydrogenase [75].

Research shows that lignin may be involved in plant protection against stresses [72]. Phenolic compounds cover a wide range of compounds, such as phenolic acids and flavonoids [76]. The methylation of caffeic acid yields ferulic acid, which forms lignin precursors with *p*-coumaric acid. Finally, lignin is synthesized from the polymerization of *p*-coumaric, ferulic, and sinapic acids, and their alcohol. Lignin accumulation and early lignification of roots have been proven in metal stress conditions. Lignification has a protective role in plants and hinders the penetration of harmful metals [77].

Glomalin was first described in 1996 as a glycoprotein secreted by AMF spores and hyphae. It was named glomalin as this protein is secreted by the genera *Acaulospora, Entrophospora*, *Gigaspora*, *Glomus*, and *Scutellospora* from the order of Glomales. Glycoproteins are stable before heat and can survive in the soil for years, resisting microbial invasions [78]. The soil proteins related to glomalin attach mineral and organic matter to soil particulates for a long time because they contain 85% polysaccharides, which are resistant to microbial decomposition [79]. The total glomalin content in the soil has strong bonds with the total organic matter content in the soil [80,81]. Glomalin is synthesized by living hyphae of obligate biotrophic AMF and its concentration depends on soil properties, weather conditions, the fungi involved, the host plants, and their productivity [82].

The negative effect of potentially hazardous elements on glomalin synthesis can partially be explained by the fact that the net photosynthesis rate of the host is strongly inhibited by trace metal pollution, resulting in low carbon deposition in the rhizosphere soil and, consequently, the retarded growth of AMF [83]. Based on the results, the glomalin content improved after the biofertilizer application, which suggests AMF as an alternative to chemical fertilizers [84]. These results are probably associated with the role of AMF in the metabolism of different compounds produced by plant roots [85]. In addition, AMF can improve the chemical and nutritional quality of the soil by various mechanisms, such as the availability of phosphorous, the structure of the soil and aggregates, and the release of glomalin [84]. Seminal research has revealed the direct relationship between soil glomalin content and its physicochemical properties [85,86,87].

Mycorrhiza (AMF) releases glomalin that contains polysaccharides, a sticky matter that enters into hydrophobic reactions with soil particulates to stick to aggregates. It also binds to Fe to form stable bridges with clay minerals [88]. It is evident in our results that glomalin was significantly higher in the treatments containing mycorrhiza. It has been found that the increase in glomalin-related soil protein is associated with the increase in soil organic carbon, available phosphorus, total nitrogen, and available potassium [89]. This is also supported by our results in regard to the treatments involving the NPK biofertilizer with mycorrhiza.

Plants possess multiple enzymes that serve as protective mechanisms against stress factors. One of them is PAL [90]. A study showed that *Basileus* strains (biofertilizers) induced the expression of defensive genes, for e.g., PAL, for stress suppression [91]. It can be stated that in our research, the treatments applied in stressful conditions increased PAL to enable the plant to cope with these conditions.

Among the various factors affecting crop productivity, potentially toxic elements stress, as a soil-related abiotic stress, plays a crucial role in causing poor yields on a global scale [92]. The pollutants emitted by the industry can cause the accumulation of potentially toxic elements, with destructive effects on plants and animals if they are disposed of in the environment with no treatment [93]. The application of plant growth regulators is an important way to alleviate the destructive effects of potentially toxic elements [94].

It is claimed that biochar-treated wastewater can be used to irrigate plants because biochar helps plants absorb fewer metals [16]. Biochar can reduce metal bioavailability by various mechanisms, like enhanced labile carbon for microbial assimilation and partly through the immobilization of metals, thereby reducing potential element toxicity in plants and soil microorganisms [16,95]. Biochar is rich in organic compounds and has a high cation exchange capacity and high surface area [16,96]. Biochar application reduces the uptake of cadmium and increases plant growth. This effect was observed in the treatment with biochar versus the control in the present study. Based on the results, the plants inoculated with mycorrhiza showed a lower rate of cadmium uptake. Mycorrhiza may reduce the uptake of unessential metals or separate metals in cell walls or intracellular parts, inducing resistance to cell damage. Our findings agree with Gao et al.’s explanation about cadmium in rice plants. These researchers reported that cadmium primarily accumulated in the pectin and hemicellulose 1 (HC1) components of the root cell wall. The mechanism of this action is that AMF inoculation led to an increase in the levels of pectin, HC1, and lignin content in the root cell wall. This increase was accompanied by elevated activities of L-phenylalanine ammonia-lyase and pectin methylesterase [97]. The cell wall plays a crucial role in the fixation of cadmium, thereby reducing its transportation from the root to the shoots. The inoculation of AMF has the potential to remodel the biosynthesis of the root cell wall and influence the characteristics of cadmium fixation [97]. Arbuscular mycorrhizal fungi compensate for the root toxicity of metals in plants by providing another pathway for nutrient uptake through the ultraradical hyphae. These hyphae provide access to a larger volume of soil to supply nutrients [48], increasing nitrogen absorption through increasing the activity of nitrogen metabolism enzymes [98], mobilizing immobile nutrients by lowering the pH of the rhizosphere [56], and also increasing the absorption rate of phosphorus in the form of polyphosphate [99]. On the other hand, the effective application of soil microorganisms (PGPRs) can help plant growth. Different genera of *Pseudomonas* and *Azotobacter* are PGPRs that improve plant growth and development in normal and stressful conditions [28]. This was clearly observed in our results, as fertilizers increased soil nutrients and improved plant resistance to stress. It was found that the application of biological and organic fertilizers reduced cadmium uptake. The use of bacteria was shown to be a flexible strategy by neutralizing the possible side effects of cadmium toxicity due to its plant growth-promoting properties [67]. In the obtained results, the roots contained more cadmium than the grains. The roots are the first part of the plant that comes in contact with soil cadmium, so their structural components may accumulate the greatest amount of cadmium in plant tissues, which is in agreement with the findings by Nogueirol et al. [100]. This is a physiological strategy by which plants fix the metal in their roots to prevent it from reaching the xylem, thereby mobilizing toward the branches and harming the photosynthesis system [101,102]. It is affected by factors that can contribute to increasing the concentration of potentially toxic elements in the soil [103]. The application of different fertilizer treatments reduced the TF from the roots to the grains. Using biological and organic fertilizer treatments, the plant roots accumulate cadmium and hinder its increase in corn grains, thereby reducing the concentration of the element in the food chain. Indeed, by accumulating cadmium in its roots, the plant can prevent its translocation to the shoots and the occurrence of toxicity [104].

Given the drought crisis, the application of wastewater in agriculture is an inescapable necessity. Based on our results, it can be concluded that the application of biofertilizers alone, or in combination, induces the secretion of secondary compounds against cadmium stress in corn plants, thereby mitigating the adverse effects.

## 4. Materials and Methods

### 4.1. Study Site

The research was conducted using pots in the climatic conditions of the Qahremanlu region in Urmia, Iran, in the 2020–2021 crop year. Urmia is located at the longitude 45°10′ E and the latitude 37°52′ N, with an elevation of 1332 m from sea level.

### 4.2. Soil Preparation and Planting

The soil and wastewater used in the research was analyzed and the results are given in Table 2 and Table 3. The soil sample was analyzed according to the various physicochemical properties, including the particle size distribution, pH, electrical conductivity, organic carbon, total nitrogen, and available phosphorus and potassium, using standard methods [105]. The chemical properties of the wastewater were determined using the methods described by Eaton et al. [106]. The pots used had a capacity of 10 kg, with a diameter of 40 cm and a depth of 50 cm. All pots were filled with soil to a height of 20 cm. Before planting, the soil and wastewater in the study site were sampled to determine their cadmium content. The soil and wastewater cadmium content were 14.9 mg/kg and 1 mg/L, respectively. The wastewater that was used to irrigate the pots was supplied from the Qahremanlu region to the greenhouse. The pots were sown with 10 seeds of the Dominate Hybrid corn variety and, after thinning, equal numbers of plants (4) were left in all pots.

### 4.3. Experimental Design

The research was conducted as a factorial experiment, based on a randomized complete block design with three replications. The first factor was biological and organic fertilizers at seven levels, namely Treatment 1: control (no fertilization); Treatment 2: bacterial biofertilizers (NPK) (1 g per pot) along with iron and zinc Barvar biofertilizers (3 mL to soak the seeds in each pot) containing *Pseudomonas putida*, *Pantoea agglomerans*, *Pseudomonas koreensis*, and *Pseudomonas vancouverensis* (prepared by Iran Green Technology Company, Iran); Treatment 3: arbuscular mycorrhiza fungi (50 g per pot); Treatment 4: *Trichoderma harzianum* fungi (1 g per pot); Treatment 5: biochar (200 g per pot) (Table 4); Treatment 6: a combination of bacterial and fungal biofertilizers; and Treatment 7: a combination of bacterial and fungal biofertilizers and biochar. The second factor was assigned to irrigation at two levels (conventional irrigation and irrigation with wastewater).

### 4.4. Measurement of Morphological Traits

The length and diameter of the cobs were assessed using a 20 cm ruler. To determine the grain yield per hectare, the cobs from all three replications (pots) for each treatment were measured, and the resulting average was then multiplied by 7000. The average plant density for the cultivated cultivar was 7000 plants per hectare. To calculate the number of grains per row, plants were selected from the replications for each treatment, and the grains on the rows were counted and averaged for each treatment.

### 4.5. Measurement of Total Phenol Content in Leaves

The total phenol content was determined with the method described by Ribarova et al. [107]. Briefly, 1 mL of the Folin–Ciocâlteu reagent and 1 mL of sodium carbonate 7% were mixed with 0.1 mL of the methanolic extract and incubated at laboratory temperature for 90 min. Then, the absorbance of the samples was read at 750 nm.

### 4.6. Measurement of Flavonoid Content in Leaves

The flavonoid content was determined by Chang et al.’s [108] method: 0.5 mL of the water extract was mixed with 1.5 mL of ethanol 95%, 0.1 mL of chloride aluminum 10%, 0.1 mL of 1 molar potassium acetate, and 2.8 mL of deionized water. The absorbance of the samples was read at 415 nm.

### 4.7. Extraction of Polyphenols

To extract the polyphenols, 2 g of the powdered sample was first mixed with 4 mL of the methanol solvent containing 1% acetic acid, and the extraction process was then carried out under ultrasonic waves for 20 min. The polyphenol compounds were isolated, detected, and quantified by high-performance liquid chromatography (HPLC) analysis, using an Agilent 1100 series device (the US) equipped with a 20 µL injection loop, a four-solvent gradient pump, a degasification system, and a column furnace (set at 25 °C). The diode array detector was set at 250, 272, and 310 nm. The isolation was performed in an octadecyl silane (ZORBAX Eclipse XDB with a length of 25 cm, an internal diameter of 4.6 mm, and a particle size of 5 µm), made by Dr. Mainsch, Germany. The data were processed using the Chemstation software package. For better isolation of the compounds, the elution program was employed for which the mobile phase was first initiated at a ratio of 10% of acetonitrile and 90% of acetic acid, 1% solution at a flow rate of 1 mL/min and reached 25% of acetonitrile + 75% of acetic acid, 1% solution at a flow rate of 1 mL/min. Then, it was increased to 65% of acetonitrile + 35% of acetic acid, 1% solution at a flow rate of 1 mL/min. The isolation time was 15 min [71].

### 4.8. Measurement of Glomalin

One gram of the soil around the roots was put into a centrifuge tube. Then, 8 mL of sodium citrate solution 20 mM (pH = 7) was added to it, and it was vortexed for 30 s. Then, it was autoclaved at 121 °C and centrifuged at 5000 RPM for 15 min. The supernatant was used for the measurement [78]. The total glomalin was extracted by 50 mM sodium citrate (pH = 8), for which 8 mL of this solution was added to the soil sample, and it was vortexed for 30 s. The next steps were similar to the simple extraction procedure. The supernatant was used to measure glomalin using the Bradford method. The Bradford reactive protein measurement is a general method for protein measurement [109].

To measure glomalin using the Bradford method, the Coomassie blue reagent was prepared by dissolving 100 mg of Coomassie blue color (G250) in 50 mL of ethanol 95%. Then, 100 mL of phosphoric acid 85% was added to it, and its volume was adjusted at 1 L by adding distilled water. The standard protein solution was also prepared from bovine serum albumin (BSA) at the rates of 10, 50, 100, 150, 200, and 250 µg per 1 mL of the sodium citrate solution. It should be noted that the standard protein was prepared using the solution that was already used to extract the solutions. To draw the calibration curve, 100 µL of each standard solution was poured into a test tube. Then, 100 µL of the sodium citrate solution was used as the control. In the next step, 5 mL of the Coomassie blue reagent was added to each tube. Immediately after adding the protein reagent, the absorbance of the samples was read at 595 nm with a spectrophotometer.

### 4.9. Measurement of Phenylalanine Ammonia-Lyase (PAL) Activity

The plant extract was prepared using an extraction buffer. The extract was mixed with measurement buffer and was kept in a hot water bath at 30 °C for 30 min. The absorbance variations were read at 290 nm. The enzyme activity unit was calculated using the extinction coefficient of cinnamic acid (9630 mM^−1^ cm^−1^) in terms of nmol *trans*-cinnamic acid mg^−1^ protein min^-1^ [110].

### 4.10. Measurement of Cadmium Content

The cadmium content was measured by the atomic absorbance method using ashes of the samples (grains and root), based on the method by Eaton and Franson [106]. The amount of cadmium was read using an Analyst 800 device, equipped with a furnace system, made by Perkin Elmer (model Analyst 800, made in America). The amount of cadmium in the soil and water were also measured using atomic absorption spectroscopy [106].

### 4.11. Translocation Factor (TF)

The TF was calculated as the ratio of the cadmium concentration in the grain to that in the root of the plant [111].

TF = C_grain_/C_root_

where C_grain_ is the cadmium concentration in the grain (mg/kg), and C_root_ is the cadmium concentration in the root (mg/kg).

## 5. Conclusions

Irrigation with wastewater influences the physical and chemical characteristics of the soil and the performance of the plant. So, wastewater and soil characteristics should be considered in the management of irrigation with wastewater for crop production. The conclusions on the morphological traits and antioxidant characteristics of the plants revealed that the conventional water + biochar + mycorrhiza + *Trichoderma* + NPK (WiA) treatment was the best treatment altogether. Also, the best treatments involving wastewater were biochar + mycorrhiza + *Trichoderma* + NPK (WwA) and mycorrhiza + *Trichoderma* + NPK (WwMTN). The findings from the analysis of the glomalin and PAL indicated that the treatment with biochar + mycorrhiza + *Trichoderma* + NPK (WwA) performed the best. The application of composite fertilizers reduced the cadmium uptake. It can be claimed that the biological and organic fertilizers used in this research can reduce the destructive effect of cadmium contained in wastewater. Using biochar and biofertilizers is a remedial, nature friendly, low cost, and promising method to rehabilitate contaminated soils by reducing the availability and absorption of potentially toxic metals and improving soil fertility and plant growth in agriculture. The application of biofertilizers involves the need to comprehend the diverse methods through which microorganisms safeguard and enhance plant development in the face of detrimental elements in the soil. These protective mechanisms may vary depending on the plant growth-promoting characteristics and cadmium-binding capacities of the biofertilizers. These findings open a new pathway for further research.

## Figures and Tables

**Table 1:** The results of the analysis of variance on the morphological and biochemical traits.

Sources of Variation	df	Cob Length (cm)	Cob Diameter (cm)	Grains/Row (Number)	Grain Yield/ha (kg ha^−1^)	Leaf Phenols (mg g^−1^ FW ^1^)	Leaf Flavonoids (mg g^−1^ FW)	Simple Glomalin (µg ml^−1^)	Total Glomalin (µg ml^−1^)	PAL ^2^ (nmCA mgpr^−1^ 30 min)	Root Cd Content (mg kg^−1^)	Grain Cd Content (mg kg^−1^)	TF ^3^
		Means of squares											
Replication	2	1.79 **	1.79 **	1.68 *	19,804.1 **	2.08 *	0.0001 ^ns^	32,011.1 ^ns^	74,240 ^ns^	0.043 ^ns^	0.002 ^ns^	0.0002 ^ns^	0.001 ^ns^
Irrigation (A)	1	63.14 **	54.85 **	49.29 **	5,367,862.5 **	7.42 **	0.0004 ^ns^	5,833,314.1 **	12,126,324 **	573.76 **	0.00005 ^ns^	0.017 **	0.0007 ^ns^
Fertilizers (B)	6	24.14 **	21.27 **	25.99 **	1,271,598.6 **	121.05 **	0.015 **	12,051,206.1 **	176,962,602 **	6203.15 **	0.194 **	0.026 **	0.566 **
A × B	6	1.56 **	0.996 **	1.01 *	84,981.94 **	7.44 **	0.002 **	13,131,508.5 **	3,727,531 **	727.51 **	0.021 **	0.006 **	0.198 **
Experimental error	26	0.21	0.98	0.39	1963.14	0.549	0.0001	100,982.6	41,855	0.045	0.001	0.0004	0.002
CV ^4^		4.53	4.29	7.23	4.48	4.36	4.58	1.77	1.89	1.77	12.05	28.08	15.60

^1^ FW—fresh weight; ^2^ PAL—phenylalanine ammonia-lyase; ^3^ TF—translocation factor from the roots to the grains; ^4^ CV—coefficient of variations; ns, **, and *, represent non-significance and significance at the *p* < 0.01 and *p* < 0.05 levels, respectively.

**Table 2:** Physico-chemical characteristics of the soil.

pH	EC ^1^ (ds/m)	Sand (g/kg)	Silt (g/kg)	Clay (g/kg)	Total N (g/kg)	Available P (mg/kg)	Available K (mg/kg)	OC ^2^ (g/kg)
8.1	1.05	130	300	570	3.4	27	235	32

^1^ EC—electrical conductivity; ^2^ OC—organic carbon.

**Table 3:** Physico-chemical characteristics of the wastewater.

pH	EC ^1^ (dS/m)	TDS ^2^ (mg/L)
7.3	1.36	820

^1^ EC—electrical conductivity; ^2^ TDS—total dissolved solids.

**Table 4:** Physico-chemical characteristics of the biochar.

N (g/kg)	P (g/kg)	K (g/kg)	EC ^1^ (ds/m)	OC ^2^ (g/kg)	pH	C/N
8.6	3.1	3.9	0.74	550	8.15	57.5

^1^ EC—electrical conductivity, ^2^ OC—organic carbon.

## Data Availability

The data that support the findings of this study are available on request from the corresponding author.

## Supplementary Materials

The following supporting information can be downloaded at: https://www.mdpi.com/article/10.3390/plants13101331/s1, Figure S1: The comparison of means for gallic acid (a) and caffeic acid (b) in plants treated with Ww (wastewater), Wi (conventional water), and fertilizers, which include C (control), Bch (biochar), M (mycorrhiza, T: *Trichoderma)*, N (NPK), MTN (mycorrhiza + *Trichoderma* + NPK), A (biochar + mycorrhiza + *Trichoderma* + NPK); Figure S2: The comparison of means for chlorogenic acid (a) and quercetin (b) in plants treated with Ww (wastewater), Wi (conventional water), and fertilizers, which include C (control), Bch (biochar), M (mycorrhiza), T (*Trichoderma)*, N (NPK), MTN (mycorrhiza + *Trichoderma* + NPK), A (biochar + mycorrhiza + *Trichoderma* + NPK); Figure S3: The comparison of means for rutin (a) and coumaric acid (b) in plants treated with Ww (wastewater), Wi (conventional water), and fertilizers, which include C (control), Bch (biochar), M (mycorrhiza), T (*Trichoderma)*, N (NPK), MTN (mycorrhiza + *Trichoderma* + NPK), A (biochar + mycorrhiza + *Trichoderma* + NPK); Figure S4: The comparison of means for rosmarinic acid (a), cinnamic acid (b), and apigenin (c) in plants treated with Ww (wastewater), Wi (conventional water), and fertilizers, which include C (control), Bch (biochar), M (mycorrhiza), T (*Trichoderma)*, N (NPK), MTN (mycorrhiza + *Trichoderma* + NPK), A (biochar + mycorrhiza + *Trichoderma* + NPK).
