# Supplementary material for: Combined Effect of Biological and Organic Fertilizers on Agrobiochemical Traits of Corn (Zea mays L.) under Wastewater Irrigation
Source: Plants (Basel). 2024 May 12;13(10):1331. doi: 10.3390/plants13101331 (PMC11124832; doi:10.3390/plants13101331)
Supplement: Supplementary file 1 [file plants-13-01331-s001.zip › plants-2857414-supplementary.pdf]

Supplementary Material

Table of Contents

Figure S1. The comparison of means for gallic acid (a) and caffeic acid (b) in plants treated with Ww: Wastewater, Wi: Conventional water, and fertilizers include: C: Control, Bch: Biochar, M: Mycorrhiza, T: *Trichoderma*, N: NPK, MTN: Mycorrhiza + *Trichoderma* + NPK, A: Biochar + Mycorrhiza + *Trichoderma* + NPK.

Figure S2. The comparison of means for chlorogenic acid (a) and quercetin (b) in plants treated with Ww: Wastewater, Wi: Conventional water, and fertilizers include: C: Control, Bch: Biochar, M: Mycorrhiza, T: *Trichoderma*, N: NPK, MTN: Mycorrhiza + *Trichoderma* + NPK, A: Biochar + Mycorrhiza + *Trichoderma* + NPK.

Figure S3. The comparison of means for rutin (a) and coumaric acid (b) in plants treated with Ww: Wastewater, Wi: Conventional water, and fertilizers include: C: Control, Bch: Biochar, M: Mycorrhiza, T: *Trichoderma*, N: NPK, MTN: Mycorrhiza + *Trichoderma* + NPK, A: Biochar + Mycorrhiza + *Trichoderma* + NPK.

Figure S4. The comparison of means for rosmaric acid (a), cinamic acid (b) and apigenin (c) in plants treated with Ww: Wastewater, Wi: Conventional water, and fertilizers include: C: Control, Bch: Biochar, M: Mycorrhiza, T: *Trichoderma*, N: NPK, MTN: Mycorrhiza + *Trichoderma* + NPK, A: Biochar + Mycorrhiza + *Trichoderma* + NPK.

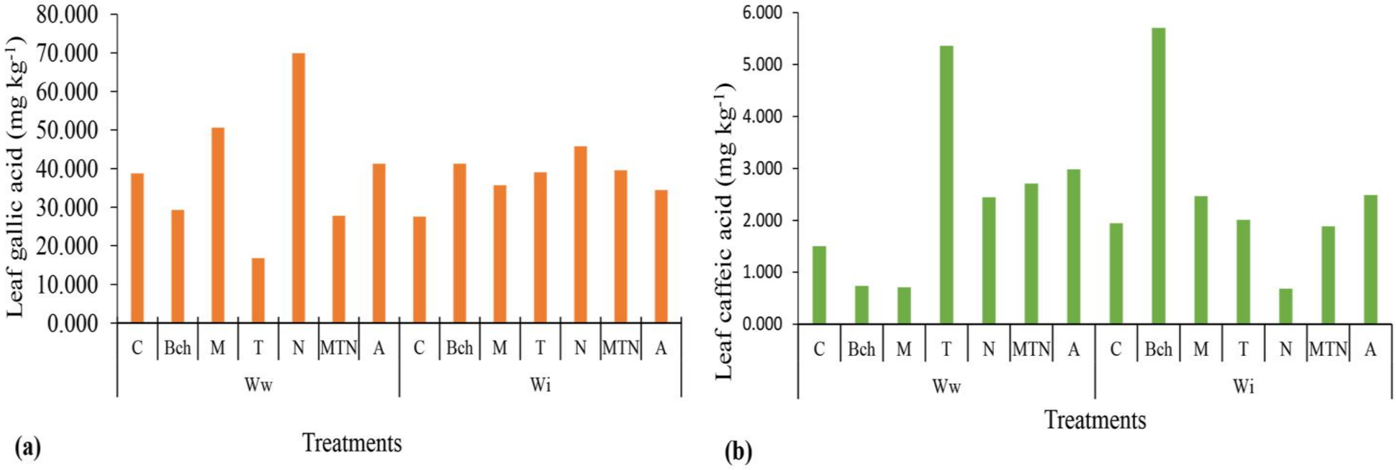

Figure S1. The comparison of means for gallic acid (a) and caffeic acid (b) in plants treated with Ww: Wastewater, Wi: Conventional water, and fertilizers include: C: Control, Bch: Biochar, M: Mycorrhiza, T: *Trichoderma*, N: NPK, MTN: Mycorrhiza + *Trichoderma* + NPK, A: Biochar + Mycorrhiza + *Trichoderma* + NPK.

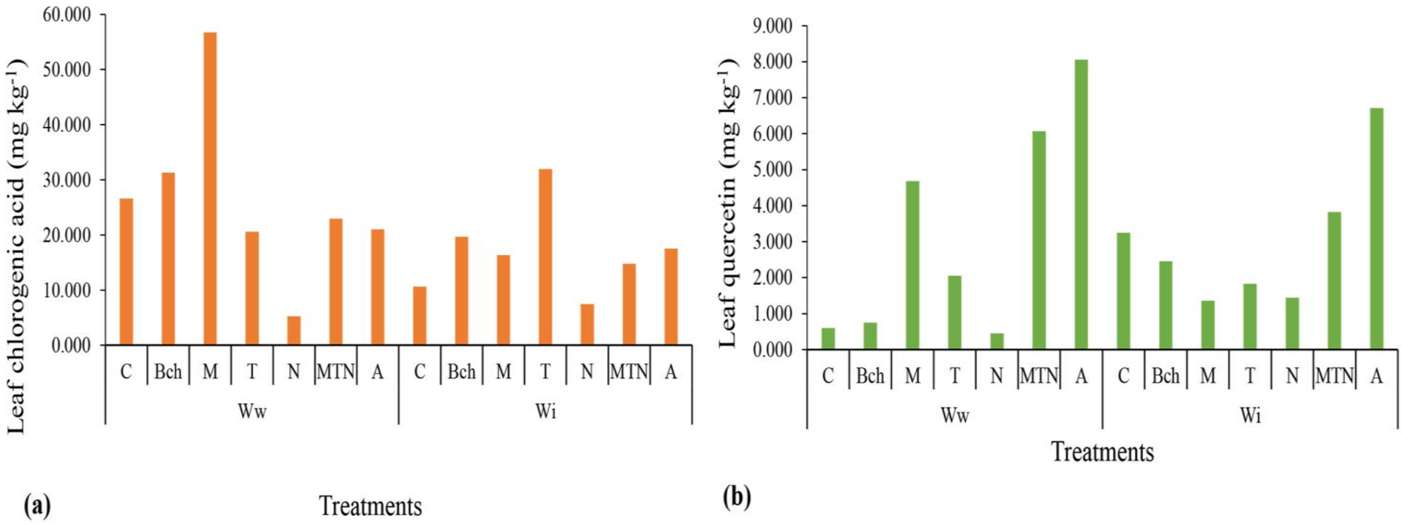

Figure S2. The comparison of means for chlorogenic acid (a) and quercetin (b) in plants treated with Ww: Wastewater, Wi: Conventional water, and fertilizers include: C: Control, Bch: Biochar, M: Mycorrhiza, T: *Trichoderma*, N: NPK, MTN: Mycorrhiza + *Trichoderma* + NPK, A: Biochar + Mycorrhiza + *Trichoderma* + NPK.

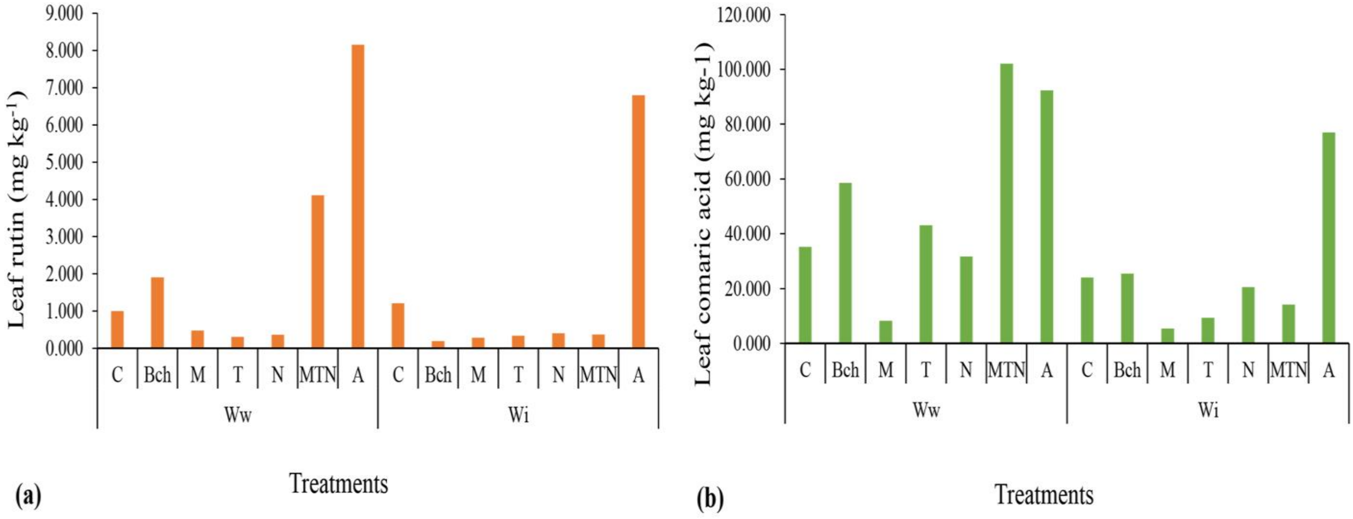

Figure S3. The comparison of means for rutin (a) and coumaric acid (b) in plants treated with Ww: Wastewater, Wi: Conventional water, and fertilizers include: C: Control, Bch: Biochar, M: Mycorrhiza, T: *Trichoderma*, N: NPK, MTN: Mycorrhiza + *Trichoderma* + NPK, A: Biochar + Mycorrhiza + *Trichoderma* + NPK.

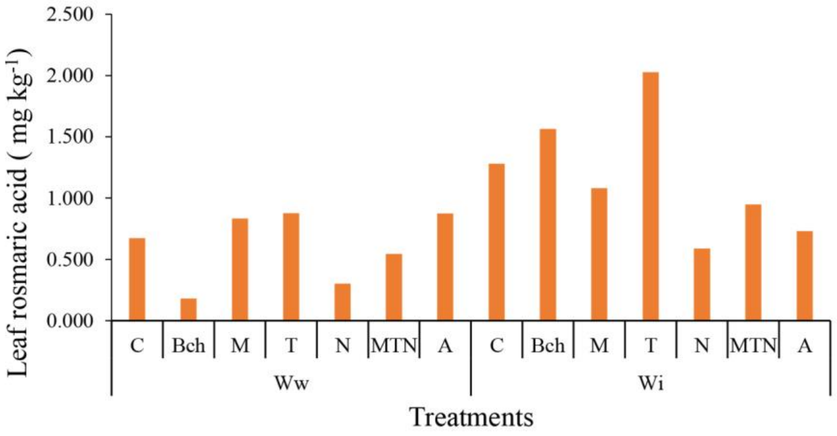

(a)

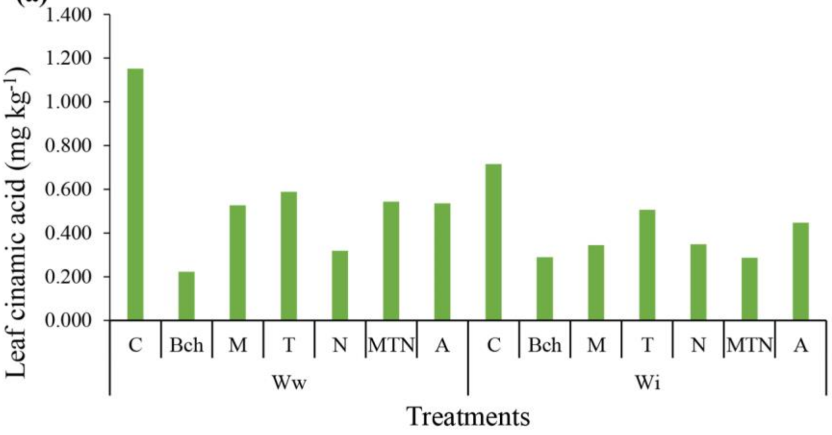

(b)

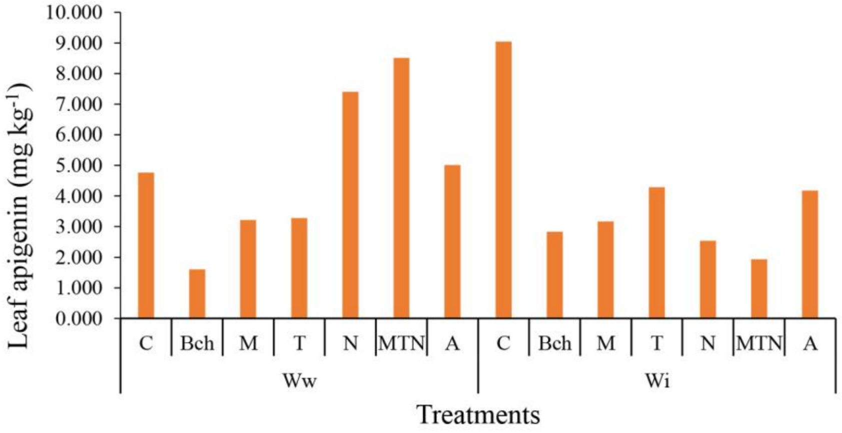

(c)

Figure S4. The comparison of means for rosmaric acid (a), cinamic acid (b) and apigenin (c) in plants treated with Ww: Wastewater, Wi: Conventional water, and fertilizers include: C: Control, Bch: Biochar, M: Mycorrhiza, T: *Trichoderma*, N: NPK, MTN: Mycorrhiza + *Trichoderma* + NPK, A: Biochar + Mycorrhiza + *Trichoderma* + NPK.
